# Long term survey of the fish community and associated benthic fauna of the Seine estuary nursery grounds

**DOI:** 10.1038/s41597-020-0572-x

**Published:** 2020-07-13

**Authors:** Thibault Cariou, Laurent Dubroca, Camille Vogel

**Affiliations:** IFREMER, Ctr Manche Mer-du-Nord, Laboratoire de Ressources Halieutiques, F-14520 Port en Bessin, France

**Keywords:** Biodiversity, Marine biology, Population dynamics

## Abstract

Estuaries are crucial ecosystems where human activities deeply affect numerous ecological functions. Here we present a survey dataset based on the monitoring of fish nursery grounds of the Seine estuary and eastern bay of Seine collected once a year using a beam trawl during three distinct periods (1995-2002, 2008–2010 and 2017–2019). The surveys happen at the start of autumn in order to maximize the catchability of juvenile fish. The beam trawl mainly targets benthic and demersal species on a study area that extends over 600 square kilometers. The dataset includes abundance and densities of 161 species for 634 hauls performed around 40 stations each year. These data can be used by fishery scientists and ecologists motivated by early life stages of commercial species or by the impact of human disturbances, such as harbor developments, on estuarine communities.

## Abstract (summary)

**Table:** 

Measurement(s)	Abundance • Population Density
Technology Type(s)	Manual Count
Factor Type(s)	year of data collection
Sample Characteristic - Organism	fish
Sample Characteristic - Environment	estuary • bay
Sample Characteristic - Location	Seine

Machine-accessible metadata file describing the reported data: 10.6084/m9.figshare.12515298

## Background & Summary

Monitoring programs for ecological purposes provide valuable information. Their interest increases as time series lengthen over the years. Marine ecosystems currently undergo significant alterations because of human activities. As such, ecological monitoring is key to assess anthropogenic impacts on marine resources and habitats. Marine historical ecology, which relies on the resulting datasets, is becoming popular^1^. The discipline expands in marine conservation and in fisheries management, promoting policies that consider ecosystems as a whole^2^.

The Seine estuary is located on the French coast of the eastern Channel. It is a very dynamic environment, where two contrasting backgrounds coexist and shape the ecosystem: intense human pressure on the one hand, and crucial ecological function on the other. Today, the Seine watershed supports an important part of the French industrial development and agricultural activity. With the presence of Paris and Rouen, the Seine River also undergoes significant demographic pressure. Human pressure has profoundly impacted the estuary’s and river’s biological communities. The Seine River was declared as “dead” in the 1960s^3^, before national measures for water quality were considered in the 1970s. For instance, the Paris Convention in 1974 aimed at protecting the ocean from land based pollution, especially substances like heavy metals and PCBs. Despite these regulations, the Seine estuary still recorded one of the highest concentrations of PCBs in mussels^4^ in 2006 compared to other regions of the globe. The Seine estuary is also a historical fishing ground for brown shrimp (*Crangon crangon*) and flatfishes, such as sole (*Solea solea*) and plaice (*Pleuronectes platessa*). An extensive fleet of fishing boats under 12 m in length was historically located in the nearby harbours of Le Havre, Honfleur, Ouistreham and Trouville. However, the industrialization pushed the estuarine system further offshore (modifications in salinity gradient and tide cycles) relocating fishing activities^5^.

The Seine estuary is a nursery area for fish. It was first described in the literature by Duval in the 1980s^6,7^. It plays a core role in the life cycle of many demersal and benthic fish^8,9^ and marine invertebrates^10,11^, among them are fish species of commercial interest. Duval focused his work on describing the size and distribution of two flatfish species, sole (*Solea solea*) and dab (*Limanda limanda*) as well as one round fish species, pouting (*Trisopterus luscus*). The concept of nursery is under constant revision; the current definition is attributed to the work of Beck and collaborators^12^. Associated with shallow waters, reduced wave exposure and physical protection such as intertidal mud flats, the nursery environment must favor protection from predation^13^, growth and survival of juveniles. By definition, juvenile stages include all developmental stages before the first maturation, until the first reproduction. Enhanced growth depends on the quantity and quality of food ressources^8^. Estuaries often qualify as nursery because their high productivity^14^ potentially drives high availability and diversity of trophic resources, which benefits juveniles’ growth rate. To date, there is a debate on whether nurseries regularly reach their maximum hosting capacity based on available trophic resources. When overpassed, juveniles would display reduced fitness parameters such as growth and survival, which would in turn regulate their abundance in a retro-control loop^15,16^. Once they reach sexual maturity, individuals tend to leave the nursery for more suitable ecosystems.

Human activities in estuaries affect the development of juvenile individuals and their survivability by impacting the nursery function^17,18^. IFREMER (the French Institute for the Exploitation of the Sea) implemented scientific cruises on coastal nursery grounds off the French coast of the Channel in the early 1990’s. The surveys aim at describing the juvenile fish population and giving an insight into ecosystems functioning in these areas^19^. The NOURSEINE survey^20^ came to existence in this context, with a first occurrence in 1995. The dataset collected from 1995 to 2019 is described in this paper. It consists primarily of density values for several taxa collected in the Seine estuary using a beam trawl for sampling. The dataset allows the exploration of changes at a community or population level in time and space. It can help understanding how the nursery function may change through time and how it is impacted by human disturbances. Scientific exploitation of earlier versions of the dataset already identified such impacts at the community level^21^ and for the sole population^22^.

## Methods

**Fig. 1:**
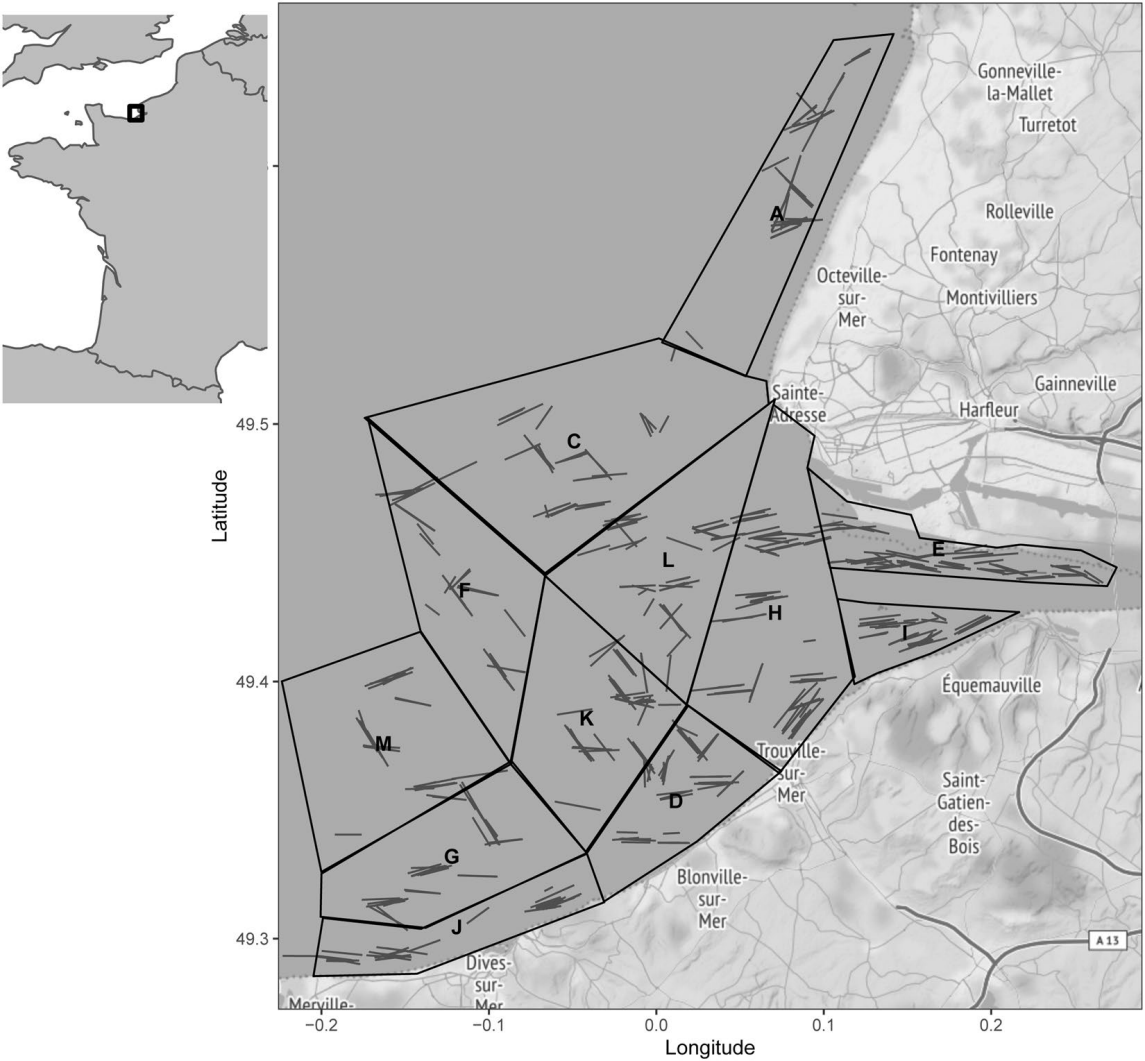
Geographical extent and sectors of the NOURSEINE survey displaying the position of hauls performed across all years. Sectors are originally established from the distance to the estuary and the bathymetry.

Data collection takes place in the Seine estuary sector extending from Ouistreham (Coordinates in projection world geodesic system 1984 or WGS84, 49°17′N 0°16′W) to Antifer (49°40′20″N 0°11′21″E) and from the Pont de Normandie (49°26′09″N 0°16′28″E) to roughly 20 meter-depth offshore to the west (Fig. 1). This 20 meter-depth limit delimitates the area considered as part of the nursery grounds^23^. The survey follows a fixed stratified sampling design. The stratification is based on bathymetry and distance to the mouth estuary. In total, 47 hauls are distributed across 12 sectors. Haul positions are randomly drawn in each sector. Due to rocky outcrop and the presence of many shipwrecks in the area, hauls’ locations are later assessed based on recommendations from professional fishers operating in the area and adjusted where needed. Morin and Schlaich^23^ provided a standardized sampling protocol for nursery zones from 1995 to 2017. In 2018, the protocol was updated in order to obtain a standardized sampling protocol on a national scale and to comply with the French Marine Strategy Framework Directive (MSFD) survey plan^19^. Differences in the two protocols for this particular survey are highlighted where needed. Sampling occurred once a year from 1995 to 2002, then from 2008 to 2010 and from 2017 to 2019. The two first periods strictly follow the first protocol. Only the last years are susceptible to changes due to protocol updates.

Sampling is carried out with a 20 mm mesh size beam trawl of 2 or 3 m wide depending on the sectors, with a 0.50 m vertical opening. The beam trawl is equipped with ground chains. Each haul lasts 15 minutes and is done against the tide at speed between 2.5 to 2.8 knots. From 2018 onward, a length of 7 minutes for the 2 m beam trawl was applied, in line with the updated national protocol. Shooting and hauling coordinates, times and depths of each haul are systematically noted. Using two different fishing gear may cause differences in the catchability of individuals, leading to differences in population characteristics estimates. An intercalibration exercise was implemented and results are presented in Riou’s work^24^. Data on flounder and sole captures were used to draw the comparison. Briefly, they showed that there were no differences in the mean density nor in the size structure for these two species. Therefore, the density values are considered comparable no matter the gear used in this protocol.

The period of reference for sampling is at the end of summer or beginning of autumn. Sampling dates scope from August 25 to September 30 over the time series. The juvenile stages here regroup individuals of age 1 and age 0. The latter corresponds to individuals who settled in the estuary on the year of the survey. Fish from age 0 group had their first period of growth over the summer. Sampling in late summer or early autumn ensures good catchability by the 20 mm mesh size beam trawl providing an accurate image of the fish distribution and abundance. Each survey day, 12 to 15 trawl stations are performed. In total, 40 to 47 stations are sampled each year. In 1996, 63 stations were surveyed as replicates were done. Hauls of a given station locate themselves relatively close to each other throughout the surveys.

After each haul, the content of the trawl is emptied on deck, and a total or partial sorting is carried out depending on the volume and homogeneity of the capture. All taxa, both fish and benthos, are sorted, identified, counted and weighted. Fishes of commercial value and all others flatfish are measured. Otoliths are collected on the main commercial fish species (sole, plaice, flounder, dab, pouting, large whiting and European bass) for later age group determination in the laboratory. In 1999, the sampling was incomplete and only commercial fish and invertebrates (King scallop and lobster) were sampled. The year was kept in the dataset to ensure continuity for these taxa.

**Fig. 2:**
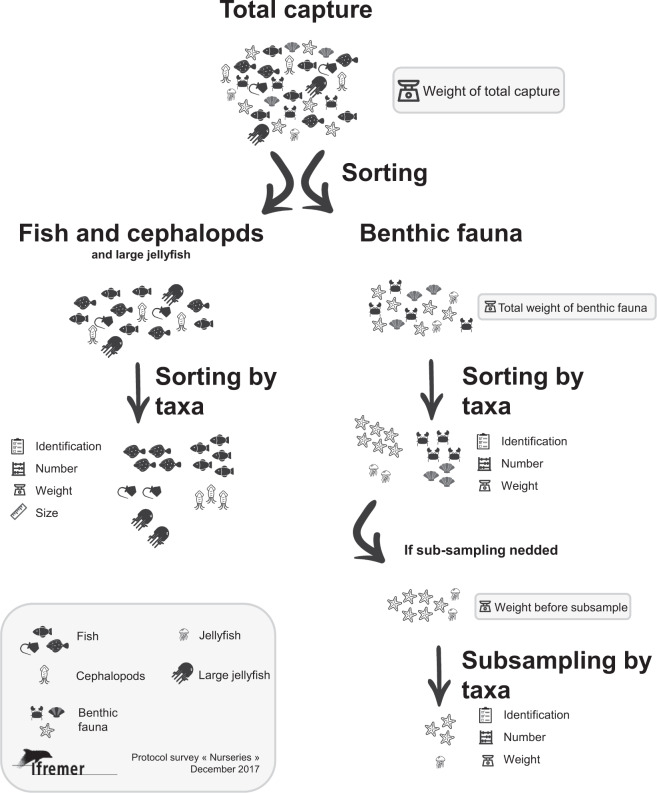
Sketch of the capture sorting process used during the NOURSEINE surveys (adapted from^19^).

Sorting the capture can be separated into three different steps (Fig. 2):

1. Total capture weighting: when the hauls are emptied on the deck, the whole capture is distributed in several baskets/box in order to weight it.
2. Fish and large taxa sorting: All fish and large taxa of invertebrates easily identified (edible crab, common spider crab, large cephalopods) are sorted, identified, numbered, measured (for fish) and weighted (total weight per taxa). Depending on the size of the capture, subsampling might be necessary. Operations are performed on the subsample in such a case. If visual identification is too difficult (for instance due to a large mud proportion), the capture is washed using a 5 mm sieve. The weight ratio between the total capture and the subsample form a “division” variable that allows the calculation of density. Another subsampling may be needed if a taxon has a high abundance. In that case, for practical reasons, only a subsample of the individuals are numbered, measured and weighted.
3. Benthic fauna sorting: What is left from the second step is weighted before the sorting operation. All taxa constituting benthic fauna are sorted, identified, numbered and weighted (total weight per taxa). Some taxa may be measured (whelk, scallop). As for step 2, a subsample might be necessary before sorting according to the quantity of benthic fauna.

All observations are manually recorded on fieldwork paper books before being checked and registered in the NOURSEINE database.

The NOURSEINE database consists of all information on fish and benthic taxa collected in a given haul, together with haul and survey information. Throughout the survey period, some changes on the level of identification are observed: while all fish taxa were normally considered and processed, sampling was reduced to commercial taxa solely during the 1999 survey. Changes in human operators may lead to mis-identifications and irregular records of a same taxa through the dataset. To provide a readily exploitable dataset, taxa clustering was applied to keep a homogenous record in the time series. Changes were mostly applied to benthic taxa (Table 1). In hauls where several taxa were clustered, abundance and weight were summed to calculate taxa density accordingly. Out of the 161 taxa initially recorded in the database, 138 remained after clustering.

**Table 1:** Outcome of the clustering process applied to homogenize the dataset.

Taxa name	Taxa clustered
*Acanthocardia* spp	*Acanthocardia echinata*
*Ammodytes* spp	*Ammodytes* sp.
	*Ammodytes tobianus*
*Ensis* spp	*Ensis magnus*
	*Ensis directus*
Gobiidae	*Gobius niger*
	Gobiidae
*Liocarcinus* spp	*Liocarcinus* sp.
	*Liocarcinus depurator*
	*Liocarcinus holsatus*
	*Liocarcinus marmoreus*
	*Liocarcinus vernalis*
*Mactra* spp	*Mactra* sp.
	*Mactra stultorum*
*Mya*spp	*Mya* sp.
	*Mya arenaria*
	*Mya arenaria*
	*Mya truncata*
Ophiuridae	*Ophiura* sp.
	*Ophiura albida*
	*Ophiura ophiura*
	Ophiuridae
*Spisula* spp	*Spisula solida*
	*Spisula subtruncata*
Inachinae	*Macropodia longirostris*
	*Macropodia linaresi*
	*Macropodia rostrata*
	*Macropodia* sp.
	*Inachus* sp.
	*Inachus dorsettensis*
Paguroidea	*Pagurus bernhardus*
	*Diogenes* sp.
	Paguroidea
	*Pagurus prideaux*
	*Pagurus cuanensis*
	*Anapagurus hyndmanni*
*Euspira* spp	*Euspira nitida*
	*Euspira catena*
Cottidae	*Taurulus*
*Chlamys* spp	*Aequipecten opercularis*
	*Mimachlamys varia*
*Doris* spp	*Doris pseudoargus*

The left column contains the taxa names as they are found in the dataset and the right column the scientific names clustered.

As only raw abundance is available first-hand, taxa densities are calculated based on trawled surface but also takes into account if the haul has been partially sorted or not. The formula to calculate the density of individuals per surface unit is:

$$Density=\frac{(Raw\,abundance\ast Division\ast Coefficient)}{Trawled\,surface}$$

where *Division* is a factor used to elevate the abundance if the whole haul was not sorted. The same formula with abundance replaced by the capture’s weight gives the captured weight per surface unit.

The database is reworked and corrected in an R script before being provided here. The coordinates of each haul are given at the beginning and the end of the fishing operation in degrees, minutes and seconds. They are converted in decimal degrees. It is in this R script that the taxa density is calculated, along with the mean weight of the capture, and that taxa clustering happens.

Efforts have been made to detect and correct any typos that potentially slipped through the first correction when data are entered in the database.

## Data Records

The data represents the densities of the different taxa encountered at hauled stations across the 14-year period where the NOURSEINE survey took place. The table contains 22435 rows and 25 columns. Following the sampling protocol prescriptions, one row corresponds either to the density of a taxa when it is only counted, or to the density of individuals of the same size within a taxa when it is counted and measured. The associated haul information is reported for each row to ensure uniqueness of the record. The community observations are published on the data depository Zenodo (10.5281/zenodo.3824354)^25^.

## Technical Validation

**Fig. 3:**
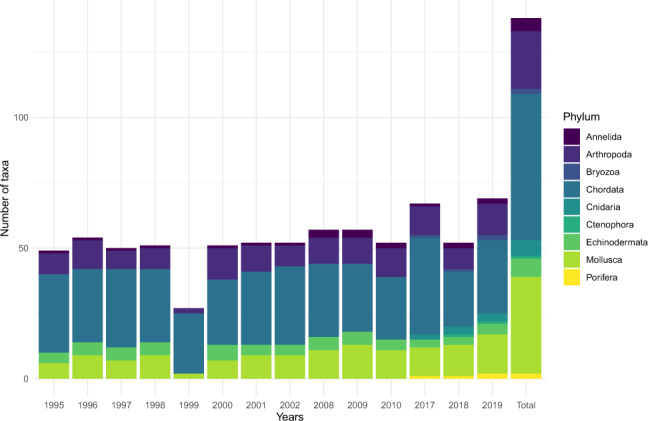
Evolution of the number of taxa (per phylum) identified during each of the 14 years of NOURSEINE surveys (between 1995 and 2019). The total gives the number of taxa identified across all years.

The taxa diversity is inspected to see the effect of different identification degrees across the survey. One hundred and thirty-eight taxa were recorded in this database (Fig. 3) mainly *Chordata*, where targeted taxa belong (i.e. flat teleostean fish). The taxa diversity is distributed mainly across three phyla: arthropods (16%), chordates (41%) and molluscs (27%). These proportions between phyla are quite stable across the period of surveys. However, there is an increase in the number of benthic fauna taxa recorded through time due to further effort put on their identification. For instance, the number of annelids taxa recorded during the surveys increased from 1.63 ± 0.80 (s.d.) from 1995 to 2002, to 3.8 ± 1.44 (s.d.) from 2008 to 2019. Likewise for the same periods of survey, the number of molluscs taxa increased from 15.56 ± 2.18 (s.d.) to 20.75 ± 2.52 (s.d.) respectively.

**Fig. 4:**
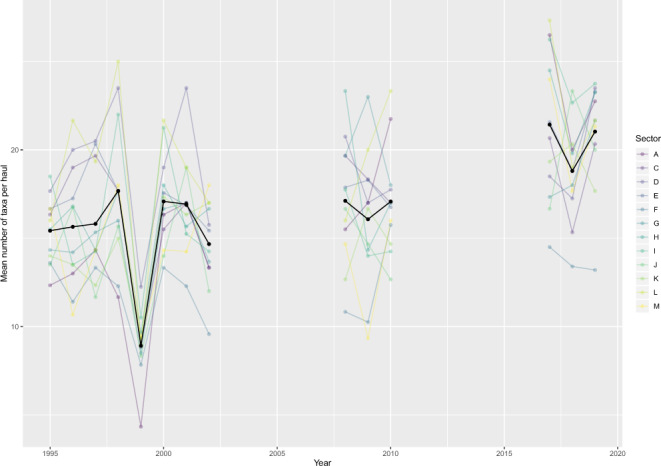
Evolution of the mean number of taxa identified in a single haul during the 14 years of surveys (between 1995 and 2019) per sector. The black line represents the evolution of the mean number of taxa calculated for all hauls across the years.

Looking at the number of taxa per haul, the distribution of all taxa is not even across the different sectors, and the richness variability is different (Fig. 4). The sectors located at the mouth of the estuary display the highest richness, whereas the sectors north and south of the navigation channel are the poorest. The majority of taxa identified and reported for the first time in the second half of the campaign (2008–2019) are benthic fauna taxa. Overall, the clustering appears to smooth the diversity enough to hide the differences in richness across the years (See figure in supplementary materials).

**Fig. 5:**
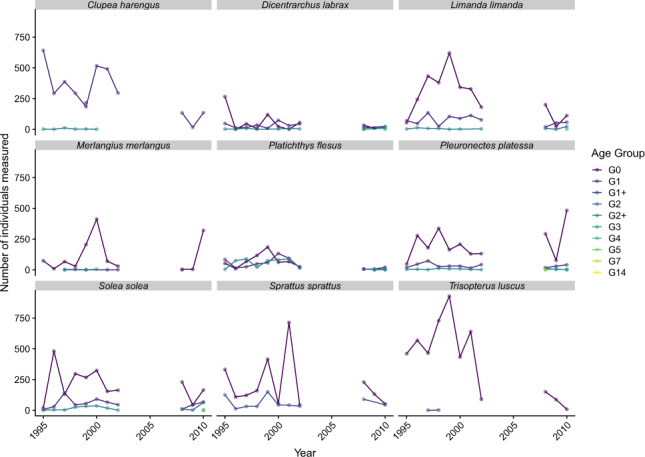
Number of individuals measured per age group for the nine species whose age is determined on more than ten cruises during the period 1995–2010.

Age, inferred from otolith readings and extrapolated through length correlation of the sampled population, is available for nine species across the period, all being taxa of commercial interest (Fig. 5). Age can be used to investigate the early life stages of those species. It indicates that the gear employed to catch fish is indeed adapted for the survey of juveniles in this nursery. Most of the individuals measured belong to the “G0” age group, which corresponds to fish that settled in the estuary on the year of the survey. For some taxa, later life stages are not recorded in the hauls because adults tend to leave this environment to go offshore. The data is heterogeneous and possesses two levels of detail according to the period considered. From 1995 to 2002, juveniles are identified and aged, but life stages after them are regrouped (Age group “G2+” or “G1+” for *Clupea harengus*). Since 2008, ages are determined for all individuals. The lack of adult individuals for some taxa makes this heterogeneity less inconvenient as the data themselves are scarce.

## Supplementary information

Supplementary material

## Data Availability

All figures have been produced using R (3.5.1) and RStudio (version 1.1.463). This script can be accessed by contacting either Thibault Cariou (thibault.cariou@gmail.com) or Camille Vogel (camille.vogel@ifremer.fr).
