# Supplementary material for: Long term survey of the fish community and associated benthic fauna of the Seine estuary nursery grounds
Source: Sci Data. 2020 Jul 13;7:229. doi: 10.1038/s41597-020-0572-x (PMC7359317; doi:10.1038/s41597-020-0572-x)

Supplementary material 1: Evolution of the number of taxa (per phylum) identified during each of the 14 years of NOURSEINE surveys (between 1995 and 2019) before taxa clustering.
The total gives the number of taxa identified across all years.

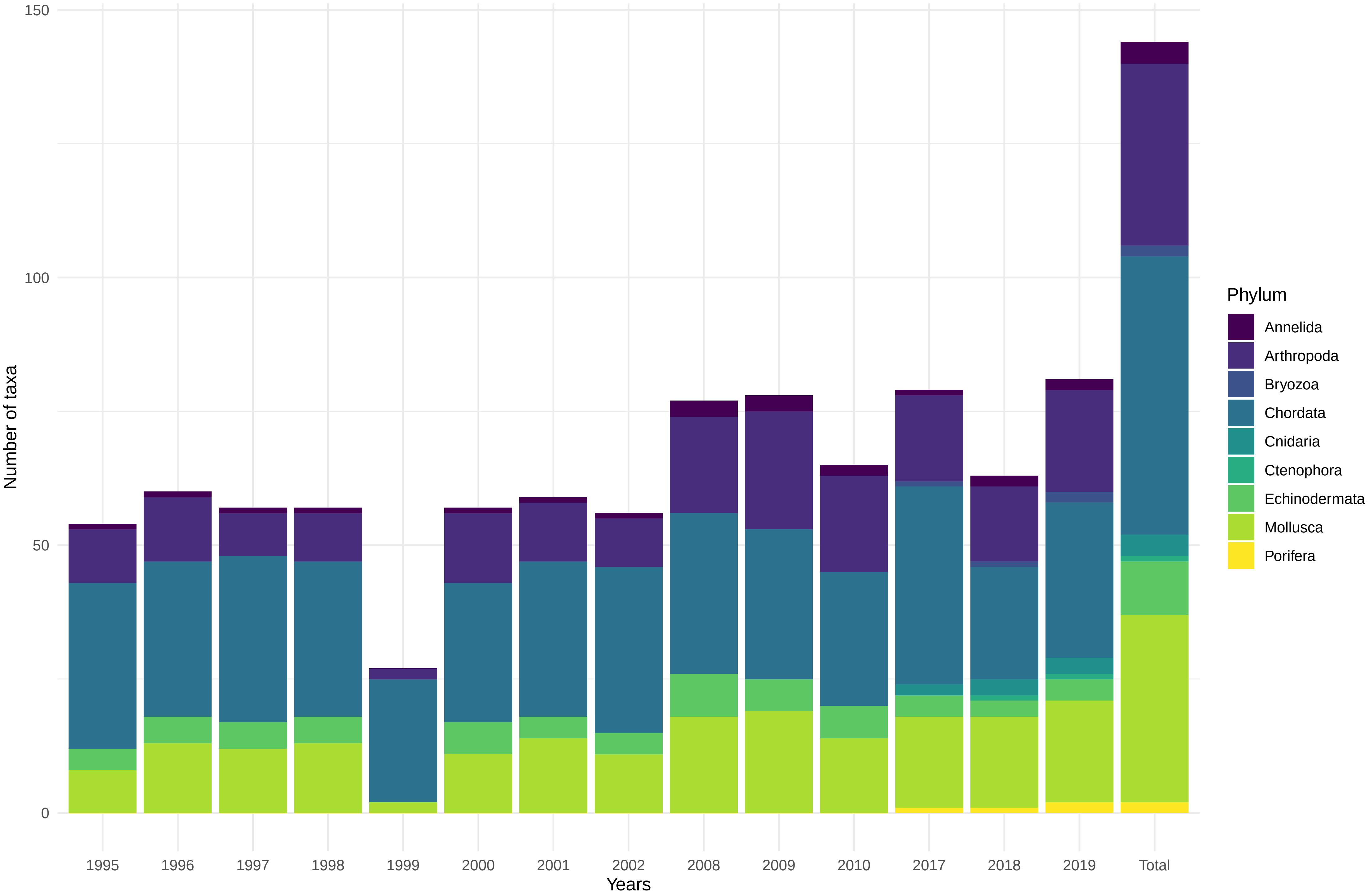

Supplement: Supplementary file 1 — Supplementary material [file 41597_2020_572_MOESM1_ESM.pdf]
